# A meta-analysis of clinicopathologic features that predict necrosis or fibrosis at post-chemotherapy retroperitoneal lymph node dissection in individuals receiving treatment for non-seminoma germ cell tumours

**DOI:** 10.3389/fonc.2022.931509

**Published:** 2022-08-17

**Authors:** Ciara Conduit, Wei Hong, Felicity Martin, Benjamin Thomas, Nathan Lawrentschuk, Jeremy Goad, Peter Grimison, Nariman Ahmadi, Ben Tran, Jeremy Lewin

**Affiliations:** ^1^ Department of Medical Oncology, Peter MacCallum Cancer Centre, Melbourne, VIC, Australia; ^2^ Sir Peter MacCallum Department of Oncology, The University of Melbourne, Parkville, VIC, Australia; ^3^ Personalised Oncology, Walter and Eliza Hall Institute of Medical Research, Melbourne, VIC, Australia; ^4^ Department of Medical Oncology, St Vincent’s Hospital Melbourne, Melbourne, VIC, Australia; ^5^ Department of Surgery, Royal Melbourne Hospital, University of Melbourne, Melbourne, VIC, Australia; ^6^ Department of Cancer Surgery, Peter MacCallum Cancer Centre, Melbourne, VIC, Australia; ^7^ Department of Medical Oncology, Chris O’Brien Lifehouse, Camperdown, NSW, Australia; ^8^ Faculty of Medicine and Health, University of Sydney, Camperdown, NSW, Australia; ^9^ Department of Urology, Chris O’Brien Lifehouse, Camperdown, NSW, Australia; ^10^ ONTrac at Peter Mac, Victorian Adolescence and Young Adult Cancer Service, Melbourne, VIC, Australia

**Keywords:** testicular neoplasms, germinoma, teratoma, pathology, meta-analysis

## Abstract

**Purpose:**

Post-chemotherapy retroperitoneal lymph node dissection (pcRPLND) for residual nodal masses is a critical component of care in metastatic testicular germ cell tumour (GCT). However, the procedure is not of therapeutic value in up to 50% of individuals in whom histopathology demonstrates post-treatment necrosis or fibrosis alone. Improved diagnostic tools and clinicopathologic features are needed to separate individuals who benefit from pcRPLND and avoid surgery in those who do not.

**Methods:**

A prospectively registered meta-analysis of studies reporting clinicopathologic features associated with teratoma, GCT and/or necrosis/fibrosis at pcRPLND for metastatic non-seminoma GCT (NSGCT) was undertaken. We examined the effect of various clinicopathologic factors on the finding of necrosis/fibrosis at pcRPLND. The log odds ratios (ORs) of each association were pooled using random-effects models.

**Results:**

Using the initial search strategy, 4,178 potentially eligible abstracts were identified. We included studies providing OR relating to clinicopathologic factors predicting pcRPLND histopathology, or where individual patient-level data were available to permit the calculation of OR. A total of 31 studies evaluating pcRPLND histopathology in 3,390 patients were eligible for inclusion, including two identified through hand-searching the reference lists of eligible studies. The following were associated with the presence of necrosis/fibrosis at pcRPLND: absence of teratomatous elements in orchidectomy (OR 3.45, 95% confidence interval [CI] 2.94-4.17); presence of seminomatous elements at orchidectomy (OR 2.71, 95% CI 1.37-5.37); normal pre-chemotherapy serum bHCG (OR 1.96, 95% CI 1.62-2.36); normal AFP (OR 3.22, 95% CI 2.49–4.15); elevated LDH (OR 1.72, 95% CI 1.37-2.17); >50% change in mass during chemotherapy (OR 4.84, 95% CI 3.94-5.94); and smaller residual mass size (<2 cm *versus >*2* cm*: OR 3.93, 95% CI 3.23-4.77; <5 cm *versus >*5* cm*: OR 4.13, 95% CI 3.26-5.23).

**Conclusions:**

In this meta-analysis, clinicopathologic features helped predict the presence of pcRPLND necrosis/fibrosis. Collaboration between centres that provide individual patient-level data is required to develop and validate clinical models and inform routine care to direct pcRPLND to individuals most likely to derive benefits.

**Systematic Review Registration:**

https://www.crd.york.ac.uk/prospero/, identifier CRD42021279699

## Introduction

Improvements in survival for individuals diagnosed with testicular germ cell tumour (GCT) have been heralded as one of the most significant advances within oncology (1, 2). GCT most commonly affects younger people, and with an ever-growing survivorship cohort, increasing attention is being placed on reducing the treatment-related morbidity given the potential wide-ranging consequences that can occur many years after cure (3, 4).

The resection of residual, post-chemotherapy masses >1 cm in marker-negative, advanced non-seminomatous GCT (NSGCT) continues to form an important part of the treatment paradigm. Owing to the risks associated with residual teratoma or viable GCT harboured within residual masses after chemotherapy, the aggressive resection of residual disease is the recommended approach in international consensus guidelines (5–8). However, post-chemotherapy retroperitoneal lymph node dissection (pcRPLND) is associated with a range of specific risks including pain, intraoperative vascular or lymphatic injury and ejaculatory dysfunction (9–12). Whilst pcRPLND is critically important in the 10%–15% and 40%–45% of individuals whose specimens contain residual viable GCT or teratoma, respectively (13, 14), improved diagnostic tools and algorithms are required to predict which individuals require pcRPLND and spare the remaining individuals with fibrosis/necrosis from unnecessary treatment from which they derive no therapeutic benefit.

Predictive models to guide decision-making regarding the appropriate selection for pcRPLND have been available for at least two decades (15–17). Clinicopathologic variables such as orchidectomy histology, particularly the presence of teratoma, pre-chemotherapy serum tumour marker levels and size of residual mass have all been reported to help predict histopathology at pcRPLND (5, 18). Accordingly, clinical models that provide weighting to each covariate have been developed (15–17). However, a lack of prospective cohorts evaluating these models has limited their use and a meta-analysis of contemporary datasets has not been performed. Thus, we performed a meta-analysis of existing literature to validate which clinicopathologic variables accurately predict pcRPLND histopathology and aid patient selection for surgery.

## Materials and methods

### Search strategy and study eligibility criteria

We searched PubMed/Medline, Embase and the Cochrane Central Register of Controlled Trials to identify studies that investigated pcRPLND histopathology in NSGCT. The search was conducted according to the Preferred Reporting Items for Systematic Reviews and Meta-Analyses (PRISMA) guidelines and was prospectively registered with the International Prospective Register of Systematic Reviews (PROSPERO) (CRD42021279699) (19). Any studies with the keywords retroperitoneal (OR) lymphadenectomy (OR) node dissection (OR) RPLND, (AND) testicular (OR) germ cell (OR) non-semin*, (AND) post-chemo* (AND) histo* (OR) viable (OR) terato* (OR) patho* were retrieved. The last search update was performed on 17 May 2022.

All studies reporting histopathological outcomes at pcRPLND for metastatic NSGCT, where odds ratios (ORs) relating to clinicopathologic variables predicting necrosis or fibrosis were either reported or able to be calculated from individual patient-level data were included in the meta-analysis.

After the removal of duplicates, two investigators screened the abstracts from each database to identify potentially eligible studies for a full-text review. Pre-defined exclusion criteria including reviews, expert opinions, case reports, irrelevant topics (i.e. pure seminoma or non-germ cell tumour, primary RPLND) and non-English language were applied during screening. The initial search yielded 4,178 articles, and after the application of exclusion criteria, 348 potentially eligible studies remained available for a full-text review.

**Figure 1:**
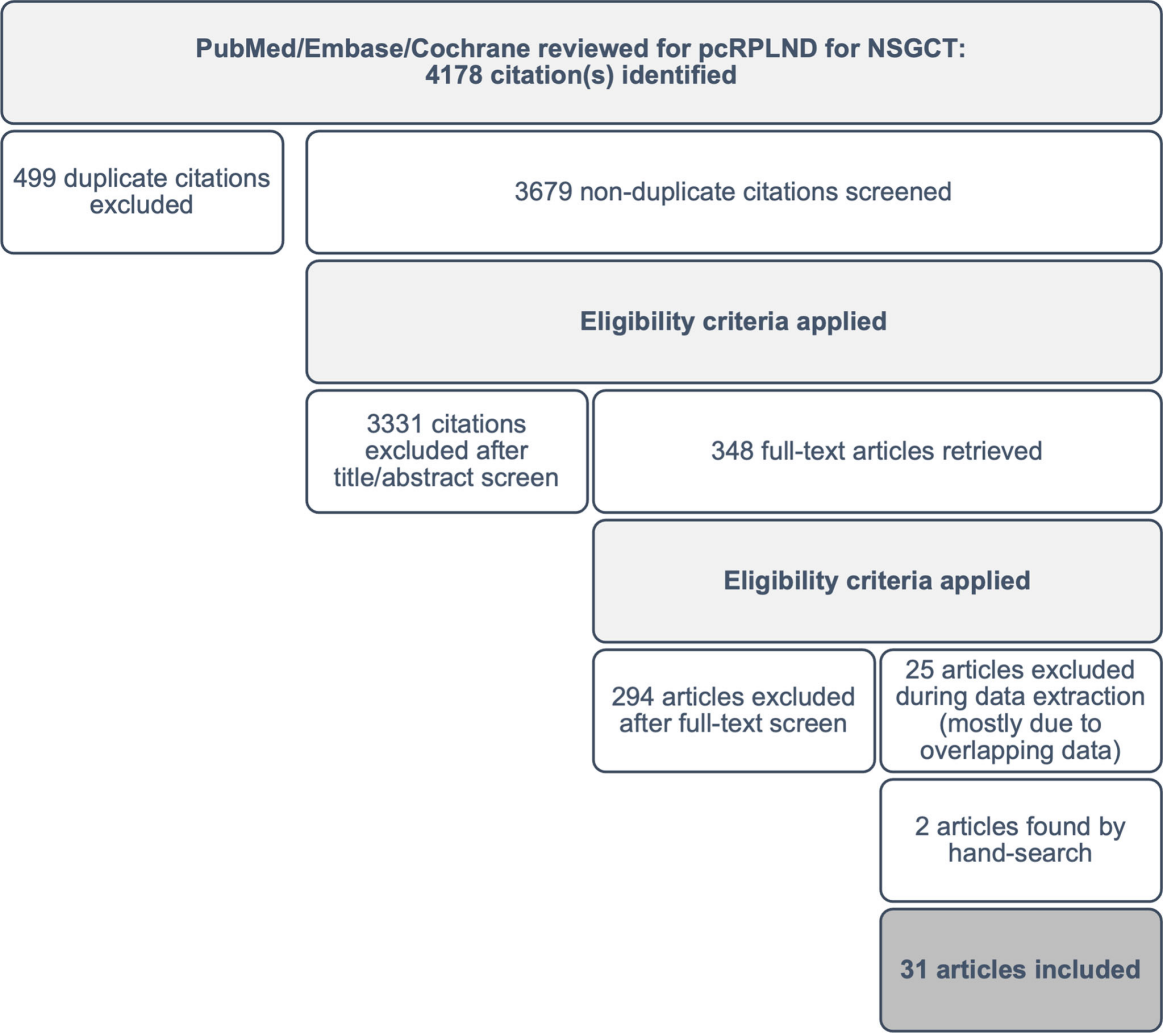
Preferred Reporting Items for Systematic Reviews and Meta-Analyses (PRISMA).

Articles that were considered potentially eligible were retrieved in full text and reviewed to confirm eligibility. Any discrepancies were discussed, and consensus reached. Reference lists from all eligible studies were surveyed to identify other potentially eligible studies that may have been missed during screening (n=2). Following a review of the full text of the 348 studies, 293 citations (84%) were excluded from the analysis. The most common reason for exclusion was an incorrect population or outcome of interest or inadequate patient-level data to permit OR calculation. An additional 25 (7%) studies were excluded during data extraction due to overlapping datasets. A total of 31 studies, including 2 sourced from the reference lists of eligible papers, were included in the meta-analysis (see **Figure 1**).

### Data extraction

For each eligible trial, we recorded in an electronic spreadsheet the first author’s name, journal, year of publication, sample size, pcRPLND histopathology and clinicopathologic variables including orchidectomy histology, disease stage, International Germ Cell Cancer Collaborative Group (IGCCCG) prognostic groups, serum tumour marker elevation [including beta-human chorionic gonadotropin (bHCG), alpha-fetoprotein (AFP) and lactate dehydrogenase (LDH)], residual mass size and change in the mass size during chemotherapy, where applicable. Where reported, we also collected pre-calculated ORs relating to predictive factors for necrosis or fibrosis at pcRPLND (with no associated teratoma or viable GCT). 95% confidence interval (CI) were reported. The risk of bias was evaluated using the Quality in Prognosis Studies tool (20) for all eligible articles across domains including participation, attrition, outcome measurement, confounding and statistical reporting.

The primary analysis comprised a pooled analysis of studies reporting or permitting calculation of ORs for clinicopathologic variables predicting necrosis/fibrosis at pcRPLND.

### Statistical analysis

We performed an aggregate patient data meta-analysis. The log OR effect estimates of each clinicopathologic factor on necrosis/fibrosis was pooled using the DerSimonian and Laird random effects model, with confidence intervals computed using the Cornfield method. Heterogeneity was estimated by comparing the result of each study with a Mantel–Haenszel fixed-effect meta-analysis result and assessed using the using the I² statistic. Publication bias was assessed using funnel plots and Egger’s test to look for small study effects. Analyses were conducted using Stata version 15.1 with the ‘metan’ and ‘metafunnel’ packages.

## Results

### Eligible studies

A total of 31 studies, including 3,390 individual patients were considered eligible for the analysis (15, 17, 21–49) (see **Table 1**). Patients were considered eligible for inclusion in the meta-analysis if they had NSGCT and underwent pcRPLND [± additional surgical resection(s)] for a residual mass, and clinicopathologic data were available. The meta-analysis of clinicopathologic variables predicting necrosis/fibrosis at pcRPLND, including orchidectomy histology, IGCCCG prognostic groups, pre-chemotherapy serum tumour marker elevation, residual mass size and other clinicopathologic features was conducted where there were two or more studies with the required information were eligible. Notably, two studies that evaluated different clinicopathologic variables in overlapping patients were included amongst these 31 papers (15, 32); however, both papers *were not* included in the same analysis of any covariate, and the larger dataset (32) formed the analysis of patient characteristics.

**Table 1:** Summary of eligible studies.

First author, Year of publication	No. of evaluable patients	Design	Years studied	Covariate analysed to predict necrosis/fibrosis at pcRPLND				
				*Orchidectomy histology*	*Pre-chemotherapy serum tumour markers*	*Residual mass size*	*Change in mass size during chemotherapy*	*IGCCCG Prognostic Group*
Comisarow, 1975 (21)	11	Retrospective	1971-1974	X				
Suurmeijer, 1984 (22)	49	Retrospective	1978-1982	X				
Jaegar, 1984 (23)	14	Retrospective	1978-1981		X			
Pizzocaro, 1985 (24)	36	Prospective	1980-1982	X				
Peckham, 1985 (25)	34	Retrospective	1977-1984	X				
Dexeus, 1989 (26)	16	Retrospective	1980-1983	X				
Sagalowsky, 1990 (27)	12	Retrospective	1979-1988	X			X	
Stomper, 1991 (28)	48	Retrospective	1979-1989	X	X		X	
Tekgul, 1994 (29)	29	Retrospective	1985-1992	X				
Matsuyama, 1994 (30)	11	Retrospective	1975-1990	X	X			
Steyerberg, 1995 (15)	555*	Meta-analysis inclusive of datasets (50–60)	1975-1993	X	X	X		
Rabbani, 1996 (31)	39	Retrospective	1985-1992	X				
Steyerberg, 2000 (32)	641*	Retrospective	1979-1996				X	
Nonomura, 2002 (33)	17	Retrospective	1995-2000	X	X			
Spermon, 2002 (34)	20	Retrospective	1998-2001	X		X		
Oldenburg, 2003 (35)	87	Retrospective	1990-2000					X
Rick, 2004 (36)	57	Retrospective	1989-1999			X		X
Vergouwe, 2007 (37)	1094	Retrospective	1977-1999	X	X	X	X	
Maldonado-Valadez, 2007 (38)	16	Retrospective	2002-2006	X		X		
Schrader, 2007 (39)	55	Retrospective	1987-2002	X				
Steiner, 2010 (40)	129	Retrospective	1984-2007	X	X	X	X	
Akbulut, 2011 (41)	15	Retrospective	2005-2010	X				
Tunio, 2011 (42)	31	Retrospective	1995-2010	X	X	X		
de Paula Miranda, 2012 (43)	30	Retrospective	2005-2011			X		
Kamel, 2016 (44)	8	Retrospective	2011-2015	X		X		
Leão, 2018 (17)	184	Retrospective	Prior to 1990-2018	X	X	X		
Öztürk, 2019 (45)	25	Retrospective	2005-2015	X				
King, 2020 (46)	57	Retrospective	2010-2016					X
Baessler, 2020 (47)	80	Retrospective	2008-2017	X				X
Taza, 2020 (48)	473	Retrospective	1990-2016					X
Malik, 2020 (49)	72	Retrospective	1994-2015					X

*Overlapping datasets, however, are not included in the same analyses of any covariates.

### Patient characteristics

Clinicopathologic factors were reported variably between studies. Of the 3,390 eligible patients, the reported age range at pcRPLND was 6–71 years old. The IGCCCG prognostic group was recorded in 1,033 (30%) patients, and 605 (59%), 211 (20%) and 217 (21%) participants had IGCCCG good-, intermediate- and poor-risk disease, respectively. Staging at enrolment was documented in a minority of participants only (10%). Of those where serum tumour markers were reported, AFP and bHCG were elevated prior to chemotherapy in at least 68% and 63% of patients, respectively.

Histopathology at orchidectomy and pcRPLND was variably documented, with some studies reporting the presence or absence of histologic subtypes within mixed tumours and others reporting predominant histologic subtypes only or grouping histologic subtypes, for example, NSGCT, not otherwise specified (NOS), non-teratoma NSGCT or simply not necrosis/fibrosis. Overall, orchidectomy histology was described in 3,290 (97%) patients, with teratoma being the most reported histologic subtype (n=1,507, 46%), followed by NSGCT, NOS (n=1,110, 34%). The most common histopathology reported at pcRPLND was necrosis/fibrosis (n=1,352, 40%), with a relative minority of patients having teratoma (n=1,107, 33%) or viable tumour reported (n=238, 7%) within the specimen.

All participants received chemotherapy prior to RPLND; however, the line of treatment, number of cycles, and choice of chemotherapy was variably documented between studies. Of those where the line of treatment was documented, 94% of participants received first-line chemotherapy prior to surgery (n=965). Where chemotherapy was documented, bleomycin, etoposide, cisplatin (BEP) was the most common regimen (n=321, 56%).

### Orchidectomy histology and post-chemotherapy retroperitoneal lymph node dissection histopathology

**Figure 2:**
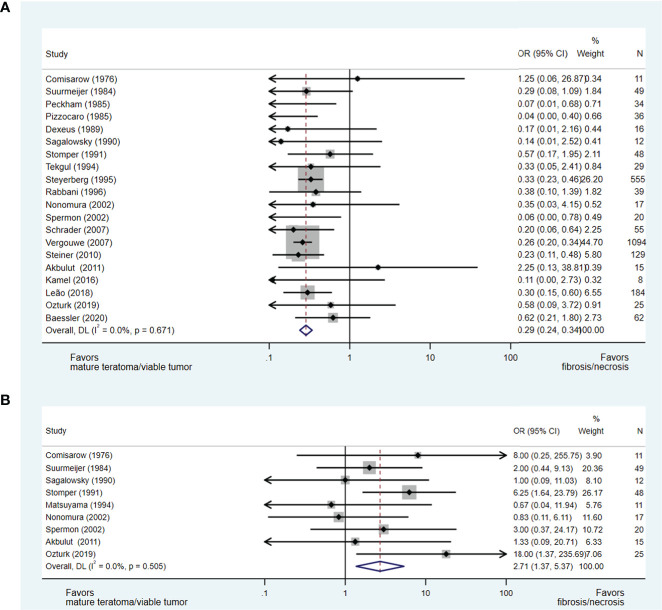
Forest plot of eligible studies evaluating relationship between presence of teratomatous elements **(A)** and seminoma **(B)** within orchidectomy and necrosis/fibrosis at pcRPLND.

Twenty-three studies (15, 17, 21, 22, 24–31, 33, 34, 37–42, 44, 45, 47) evaluating the impact of orchidectomy histology on pcRPLND histopathology were included in the analysis, including a meta-analysis of relevant studies (50–60). The presence of teratomatous elements within the orchidectomy sample was analysed in 20 studies (15, 17, 21, 22, 24–29, 31, 33, 34, 37, 39–41, 44, 45, 47), and 2,438 patients did not predict necrosis/fibrosis at pcRPLND (OR 0.29, 95% CI 0.24-0.34) over residual teratoma or viable GCT at pcRPLND (see **Figure 2A**). The presence of seminoma at orchidectomy (as a component of mixed GCT) was evaluated in nine studies (21, 22, 27, 28, 30, 33, 34, 41, 45) and 208 patients and was also shown to be predictive of necrosis/fibrosis at pcRPLND (OR 2.71, 95% CI 1.37-5.37) (see **Figure 2B**).

In contrast, necrosis/fibrosis at pcRPLND could not be reliably predicted over residual teratoma or viable GCT by the presence of other histopathologies within the orchidectomy sample, including embryonal carcinoma (OR 0.87, 95% CI 0.46-1.66) (17, 28–30, 33, 34, 42, 45) or yolk sac tumour (OR 1.25, 95% CI 0.62-2.50) (22, 28, 30, 33, 34, 38, 41, 42, 45), where there was no significant association between these variables and necrosis/fibrosis at pcRPLND (see **Supplements 1A- B**).

### Pre-chemotherapy serum tumour marker level and pcRPLND histopathology

**Figure 3:**
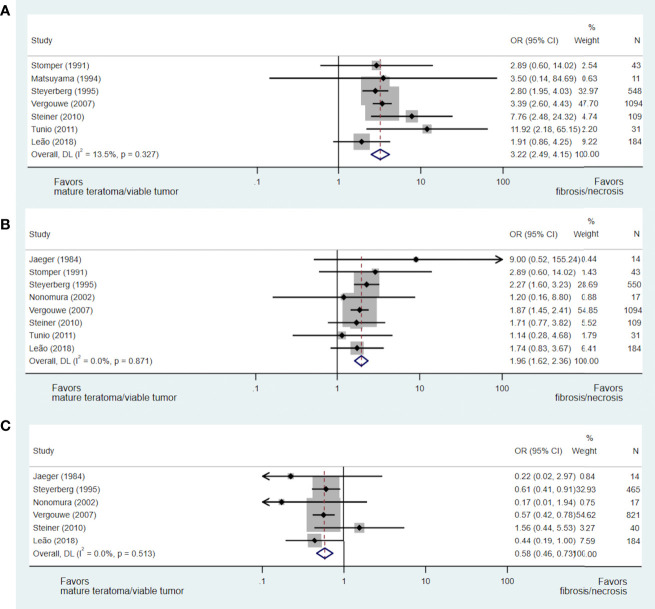
Forest plot of eligible studies evaluating relationship between normal AFP **(A)**, bHCG **(B)** and LDH **(C)** and necrosis/fibrosis at pcRPLND.

A total of nine studies including 2,053 patients addressed the issue of the predictive value of pre-chemotherapy serum tumour marker levels on necrosis/fibrosis at pcRPLND (15, 17, 23, 28, 30, 33, 37, 40, 42), including the aforementioned meta-analysis (15). Necrosis/fibrosis was significantly more likely to be identified in post-chemotherapy residual masses when compared with teratoma or viable GCT when AFP (OR 3.22, 95% CI 2.49-4.15) (15, 17, 28, 30, 37, 40, 42) or bHCG (OR 1.96, 95% CI 1.62-2.36) (15, 17, 28, 33, 37, 40, 42) were normal at the commencement of chemotherapy. In contrast, normal LDH prior to chemotherapy was negatively associated with this necrosis/fibrosis at pcRPLND (OR 0.58, 95% CI 0.46-0.73) (15, 17, 23, 33, 37, 40) (see **Figures 3A–C**).

### Change in mass size during chemotherapy and histopathology

A reduction in mass size during chemotherapy was evaluated consistently in five studies as a predictor of residual mass histopathology (27, 28, 32, 37, 40), with each study permitting the development of an OR of less than or greater than 50%, 70% and/or 90% reduction in mass size during chemotherapy.

**Figure 4:**
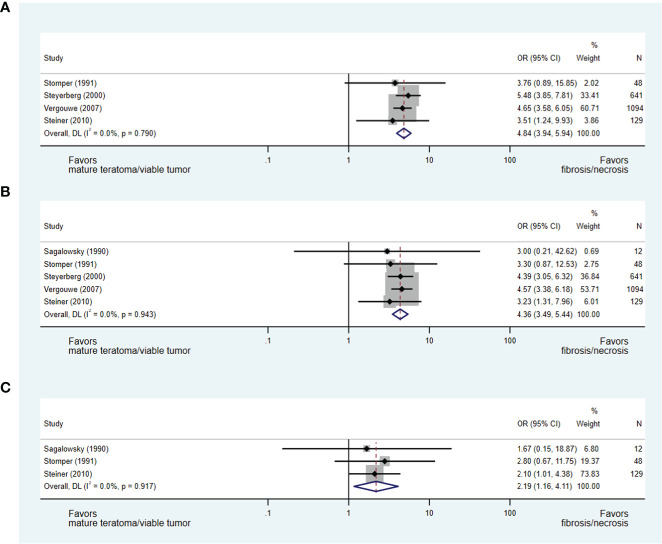
Forest plot of eligible studies evaluating relationship between ≥50% **(A)**, ≥70% **(B)** and ≥90% **(C)** change in mass size during chemotherapy and necrosis/fibrosis at pcRPLND.

In an analysis of four studies (28, 32, 37, 40), which addressed the predictive value of less than or greater than 50% reduction in mass size during chemotherapy and included 1,912 patients, a greater than 50% reduction in mass size was strongly predictive of necrosis/fibrosis over residual teratoma or viable GCT when compared to those with less than 50% reduction in mass size (OR 4.84, 95% CI 3.94-5.94) (see **Figure 4A**). Similarly, in the analyses of studies evaluating less than or greater than 70% (27, 28, 32, 37, 40) and 90% (27, 28, 40) reduction in mass size, masses undergoing greater change were more likely to represent necrosis/fibrosis than residual teratoma or viable GCT when compared to masses undergoing lesser change (≥70%: OR 4.36, 95% CI 3.49-5.44; ≥90%: OR 2.19, 95% CI 1.16-4.11) (see **Figures 4B, C**).

### Post-chemotherapy residual mass size and histopathology

The size of residual mass was evaluated in several eligible papers; however, only 10 studies utilising consistent parameters for the measurement of the residual mass were evaluable (15, 17, 34, 36–38, 40, 42–44). Each evaluable paper described the measurement of the longest transverse (axial) dimension to assign patients to groups defined as: less than or greater than 2 cm and less than or greater than 5 cm. Notably, Vergouwe et al. (37) included 136 (12%) patients proceeding to pcRPLND with residual masses <1 cm.

**Figure 5:**
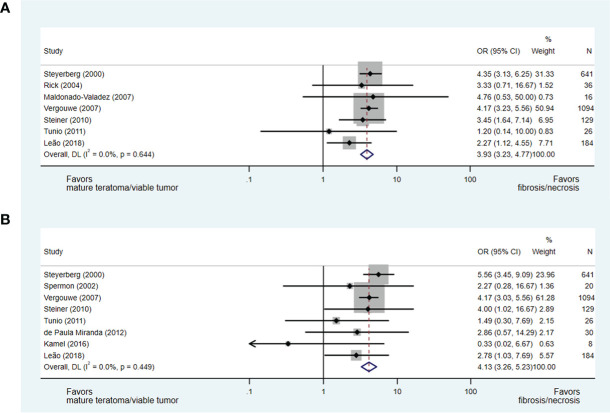
Forest plot of eligible studies evaluating relationship between residual mass size <2cm **(A)**, <5cm **(B)** and necrosis/fibrosis at pcRPLND.

Ultimately, in an analysis of seven studies (15, 17, 36–38, 40, 42), which addressed the predictive value of a residual mass size of less than or greater than 2 cm for necrosis/fibrosis and included 2,126 patients (including 6% with post-chemotherapy residual masses <1 cm), a residual mass size of <2 cm was significantly associated with necrosis/fibrosis over residual teratoma or viable GCT when compared with residual masses measuring >2 cm (OR 3.93, 95% CI 3.23-4.77). When a residual mass size of less than or greater than 5 cm was analysed in eight studies and 2,132 patients (15, 17, 34, 37, 40, 42–44), a residual mass of <5 cm was also predictive of necrosis/fibrosis at pcRPLND (OR 4.13, 95% CI 3.26-5.23) (see **Figures 5A, B**).

### International Germ Cell Cancer Collaborative Group prognostic group and post-chemotherapy retroperitoneal lymph node dissection histology

The predictive value of IGCCCG prognostic groups for pcRPLND necrosis/fibrosis was evaluable in six studies of 794 patients (35, 36, 46–49) (see **Supplement 2**). There was no significant association of necrosis/fibrosis at pcRPLND between IGCCCG good-risk NSGCT compared to IGCCCG intermediate-risk (OR 0.97, 95% CI 0.48-1.97), poor-risk (OR 0.72, 95% CI 0.40-1.31), and intermediate- or poor-risk disease (OR 1.12, 95% CI 0.66-1.93).

### Studies of heterogeneity and publication bias

Apart from the analyses of the relationship between pcRPLND histopathology and the IGCCCG prognostic group, which revealed moderate heterogeneity (I^2 =^ 35%–52%), there was minimal heterogeneity amongst the eligible studies in other analyses (I^2 =^ 0%–13.5%). Small-study effects were not detected for any pooled analyses (see **Supplement 3**).

## Discussion

The aggressive resection of residual masses in advanced NSGCT represents an integral component of treatment for many patients (5). pcRPLND shields them from risks associated with growing teratoma syndrome, late relapse, or progressive malignancy; however, pcRPLND exposes up to half of patients with post-chemotherapy residual masses to the short- and long-term hazards of surgery with no therapeutic value (13, 14). While clinicopathologic factors predicting pcRPLND histopathology have been published (5, 18), this is the first meta-analysis to comprehensively summarise contemporary literature of the value of clinicopathologic variables in predicting necrosis/fibrosis.

### Principal findings

We confirmed several key clinical findings reported in earlier studies (5, 18). Clinicopathologic factors associated with necrosis/fibrosis rather than teratoma or viable tumour within the residual mass at pcRPLND included: the absence of teratomatous elements in orchidectomy; a greater change in mass size during chemotherapy and a smaller size of post-chemotherapy residual mass; seminoma as a component of a mixed GCT resected at orchidectomy; and normal serum bHCG and AFP at the commencement of chemotherapy. The single clinicopathologic variable most predictive of necrosis/fibrosis was a >50% change in mass size during chemotherapy, which was 4.8× more likely to contain necrosis/fibrosis than teratoma/viable tumour at pcRPLND, when compared to masses undergoing <50% change during chemotherapy. Notably, the IGCCCG prognostic group did not significantly interact with pcRPLND histopathology. This has been identified previously in the abstract form where IGCCCG prognostic groups had little effect in predicting the pcRPLND pathology in a retrospective German series of 392 patients (61). Whilst some studies reported the presence of lymphovascular invasion (17, 62) in the orchidectomy sample as an additional predictor of pcRPLND histopathology, we were unable to include it in our meta-analysis as insufficient patient-level data were available to permit the calculation of OR for these covariates. The incorporation of craniocaudal lymph node length (63) as a predictor of pcRPLND histopathology was also not possible based on eligible studies; however, it would be important in the design of prospective studies of this issue.

### Strengths and limitations of the study

Our study has several strengths. Firstly, the meta-analysis was conducted using a prospectively registered protocol and in accordance with PRISMA guidelines. Secondly, it incorporated citations from the three most comprehensive databases within the field and it is unlikely that citations have been inadvertently excluded from consideration. Finally, the analysis represents data from 3,390 eligible patients and is the largest analysis of clinicopathologic variables predicting pcRPLND histopathology in NSGCT, providing a platform to propel ongoing research in this area.

However, we acknowledge a number of limitations. Firstly, as the eligible studies were almost exclusively retrospective in their design, confounding variables, such as the choice of chemotherapy regimen and number of cycles was not routinely captured. Additionally, many studies evaluated alternative primary endpoints whilst reporting pcRPLND histopathology and as such, there were significant amounts of missing data. Furthermore, due to changes in staging systems and treatment patterns during the lifetime of the eligible studies, the impact of the disease stage and chemotherapy type on pcRPLND histopathology was unable to be evaluated. This speaks to the potential issue of heterogeneity within datasets, which has plagued the existing clinical models of this issue (5). However, heterogeneity was generally considered to be low in our analysis, except for the IGCCCG prognostic group. Additionally, the inclusion of a small number of participants with residual masses less than 1 cm (15, 37) and stage 1S disease [n=1 (46)], which lies outside current treatment recommendations, introduces possible bias. Additionally, the large dataset by Vergouwe at al (37). contributed a significant amount of weight (up to 55%) to some OR calculations exposing them to potential issues; however, funnel plots reassuringly demonstrated minimal publication bias. Studies only permitting the calculation of the predictive value of clinicopathologic variables for teratoma and not necrosis/fibrosis were also excluded, given the limited clinical relevance where the treatment of alternate histopathology is strictly divergent.

### Implications for clinicians

Whilst our study evaluated almost 3,400 patients and was able to identify several clinicopathologic factors that predict necrosis/fibrosis, insufficient patient-level data were a recurrent barrier to study inclusion and will be required to aggregate clinically relevant models in the future. In rare tumours like testicular cancer, collaborative networks are needed to transform care. Existing clinical models have evaluated upwards of 1,500 patients cumulatively (15, 17, 37) and offer high specificity (>96%) and concordance (C-index >0.7) when predicting benign histology (necrosis/fibrosis) at pcRPLND. Unfortunately, each model has problems with diagnostic accuracy with false negatives, which may relate to the inclusion of heterogeneous populations recruited and analysed over several decades while the patterns of care change (5, 64). Additionally, these models lack prospective validation and clinical guidelines largely have not included them and continue to recommend the resection of all post-chemotherapy residual masses >1 cm in NSGCT provided that serum tumour markers are normal or plateauing (6–8). Where patients receiving treatment for testicular cancer will, on average, gain three decades of life following curative treatment (1), the risk of misclassifying an individual’s risk of residual teratoma or viable GCT is significant. Collaboration between leading centres treating testicular cancer will be required to move this forward in the coming years.

Meanwhile, pcRPLND continues to be a cornerstone of management for patients with NSGCT with residual masses (65, 66) erring on the side of over-treatment and preservation of cure. Historically, an open pcRPLND ensuring complete resection within a short time frame from the end of chemotherapy has been the preferred approach; however, robotic strategies are increasingly utilised in some centres to limit specific post-operative complications, such as the length of stay and post-operative pain and ileus (12, 67), but require an ongoing prospective evaluation to ensure equivalent oncologic outcomes. Regardless of the surgical strategy, referral to a high-volume centre specialising in pcRPLND is strongly recommended (8) to aid in clinical decision-making, limit potential complications, and improve the chances of a cure.

Newer techniques are required to allow a safe de-escalation of therapies in testicular cancer with a view to directing treatment towards those most likely to yield benefits and protecting the quality of life in survivors (2). One potential approach in the evaluation of post-chemotherapy residual masses in advanced NSGCT (68) is the use of microribonucleic acids (miRNAs). miR-371a-3p, -373-3p and -367-3p have been evaluated and demonstrate relatively higher serum levels when residual viable tumour was identified at pcRPLND. Unfortunately, in the same analysis, miR-371a-3p was unable to differentiate between necrosis/fibrosis and teratoma, which classically expresses lower levels of miR-371 compared to other testicular cancer subtypes, leaving an area of clinical need (69), and the studies of miR-375 have also yielded variable results (70, 71). No studies of miRNA were eligible for our analysis, and research into other candidate biomarkers is also underway (72–75).

In summary, this meta-analysis of clinicopathologic variables predicting necrosis/fibrosis at pcRPLND in NSGCT has confirmed the presence of important clinicopathologic factors, which may assist in directing personalised treatment to patients, whilst other technologies are awaited. A collaboration between key international research groups to contribute individual patient-level data, as well as the inclusion of novel molecular techniques, is required to develop and prospectively validate a clinical model for use in the clinic.

## Data availability statement

The raw data supporting the conclusions of this article will be made available by the authors, without undue reservation.

## Author contributions

All authors made substantial contributions to the conceptionand design of the work. CC, WH and FM made substantial contributions to the acquisition, analysis and interpretation ofdata. CC was responsible for the drafting the manuscript. JL provided critical supervision and guidance to conception,acquisition, interpretation and drafting. All authors provided critical revision for important intellectual content and approval of the final version to be submitted.

## Conflict of interest

BTr reports grants and personal fees from Amgen, grants and personal fees from Astra Zeneca, grants from Astellas, grants and personal fees from BMS, grants and personal fees from Janssen, grants and personal fees from Pfizer, grants and personal fees from MSD, grants and personal fees from Ipsen, personal fees from IQVIA, personal fees from Sanofi, personal fees from Tolmar, personal fees from Novartis, grants and personal fees from Bayer and personal fees from Roche, outside the submitted work.

The remaining authors declare that the research was conducted in the absence of any commercial or financial relationships that could be construed as a potential conflict of interest.

## Publisher’s note

All claims expressed in this article are solely those of the authors and do not necessarily represent those of their affiliated organizations, or those of the publisher, the editors and the reviewers. Any product that may be evaluated in this article, or claim that may be made by its manufacturer, is not guaranteed or endorsed by the publisher.

## Supplementary material

Supplement 1 Forest plot of eligible studies evaluating relationship between presence of embryonal carcinoma **(A)** and yolk sac tumour **(B)** within orchidectomy and necrosis/fibrosis at pcRPLND.

Supplement 2 Forest plot of eligible studies evaluating relationship between IGCCCG **(A)** good- versus intermediate-risk, **(B)** good- versus poor-risk and **(C)** good- versus intermediate- or poor-risk and necrosis/fibrosis at pcRPLND.

Supplement 3 Funnel plots of publication bias in analyses of **(A)** primary teratoma, **(B)** primary seminoma, **(C)**, normal pre-chemotherapy AFP, **(D)** normal pre-chemotherapy bHCG, **(E)** normal pre-chemotherapy LDH, **(F)** greater than or equal to 50% change in mass during chemotherapy, **(G)** greater than or equal to 70% change in mass during chemotherapy, **(H)** greater than or equal to 90% change in mass during chemotherapy, **(I)** residual mass size less than or greater than 2cm, and **(J)** residual mass size of less than or greater than 5cm for necrosis/fibrosis at pcRPLND.
