# Supplementary figures and images for: A meta-analysis of clinicopathologic features that predict necrosis or fibrosis at post-chemotherapy retroperitoneal lymph node dissection in individuals receiving treatment for non-seminoma germ cell tumours
Source: Front Oncol. 2022 Aug 17;12:931509. doi: 10.3389/fonc.2022.931509 (PMC9428700; doi:10.3389/fonc.2022.931509)

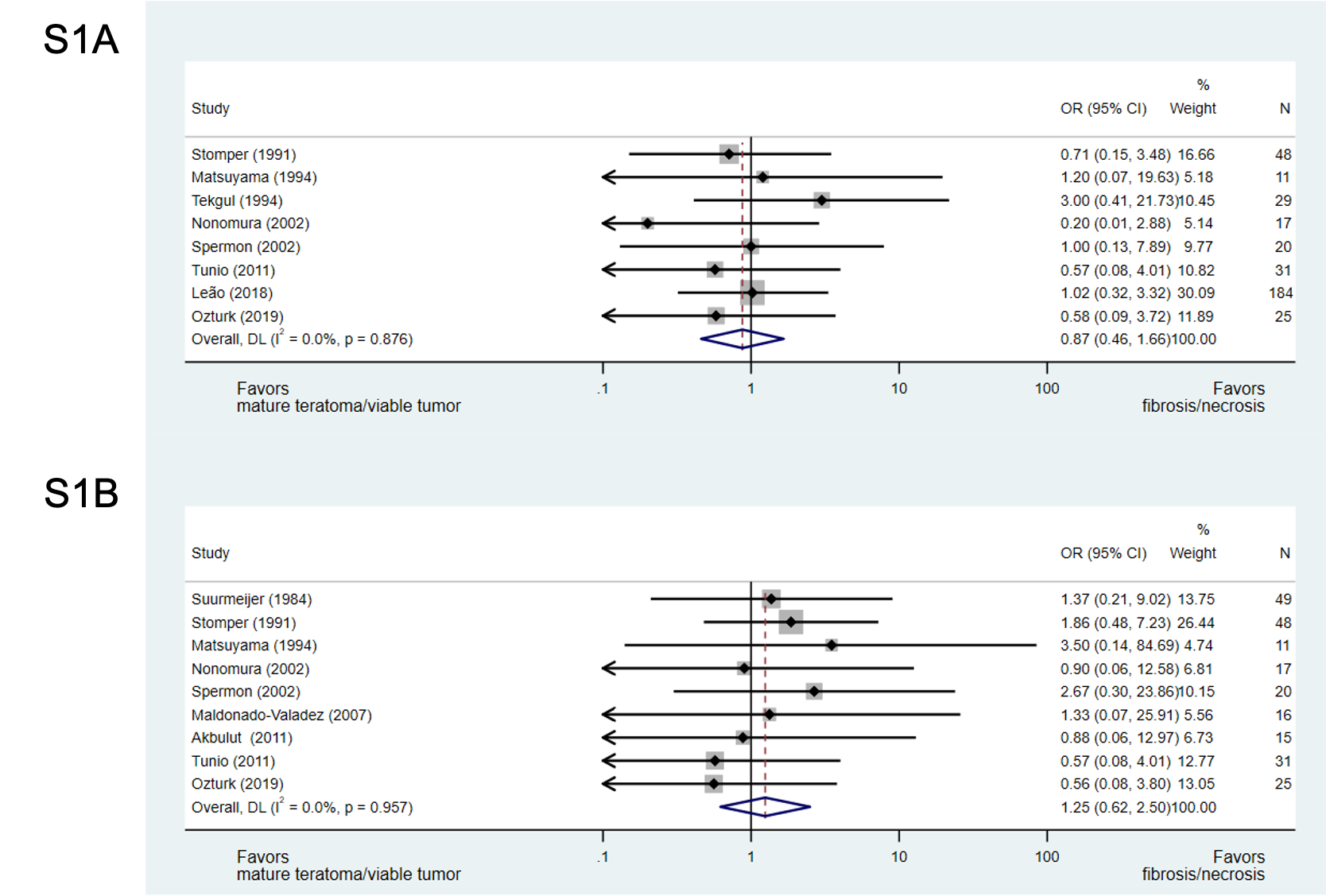

Supplement: Supplement 1 — Forest plot of eligible studies evaluating relationship between presence of embryonal carcinoma (A) and yolk sac tumour (B) within orchidectomy and necrosis/fibrosis at pcRPLND. [file Image_1.jpeg]

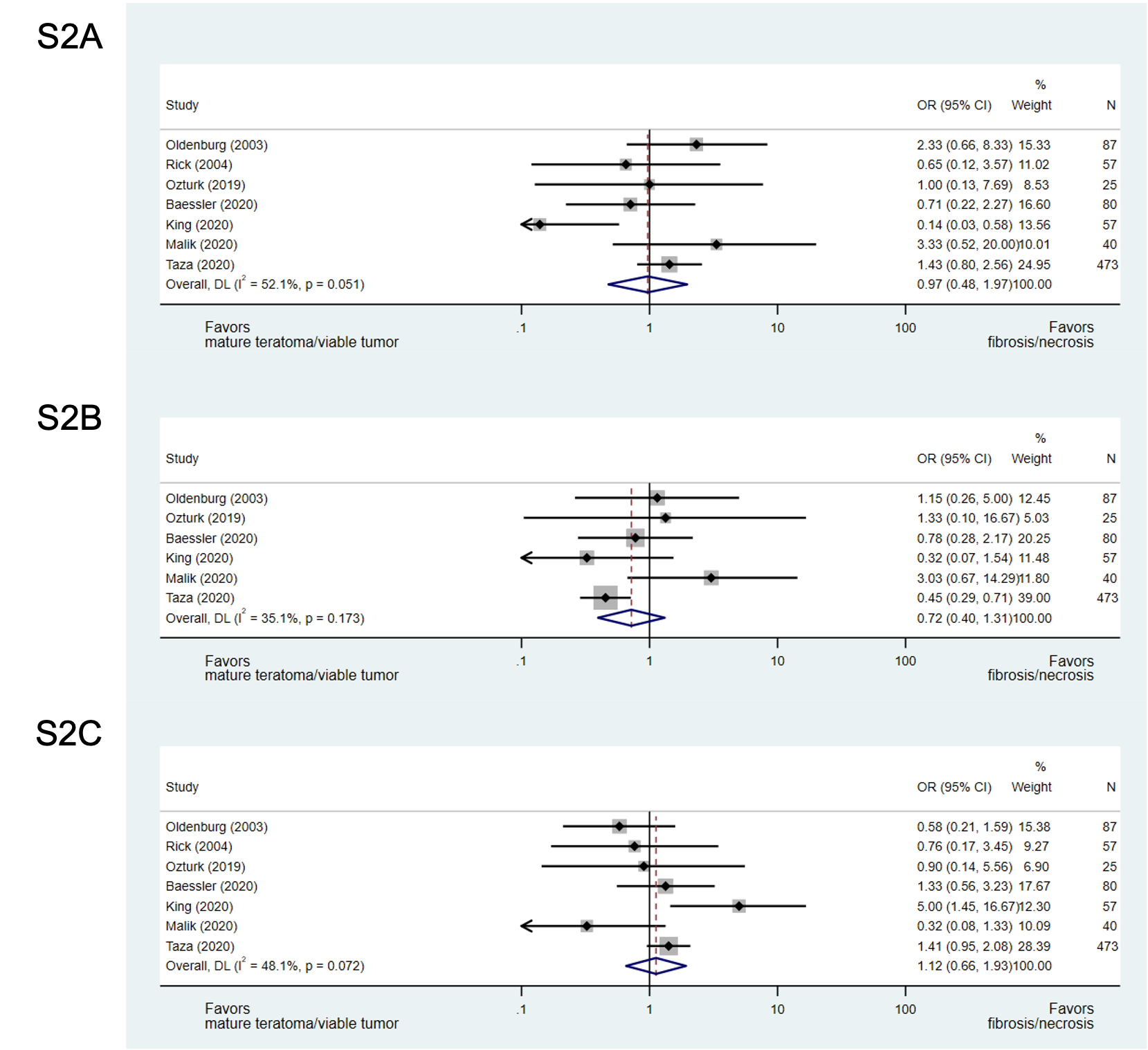

Supplement: Supplement 2 — Forest plot of eligible studies evaluating relationship between IGCCCG (A) good- versus intermediate-risk, (B) good- versus poor-risk and (C) good- versus intermediate- or poor-risk and necrosis/fibrosis at pcRPLND. [file Image_2.jpeg]

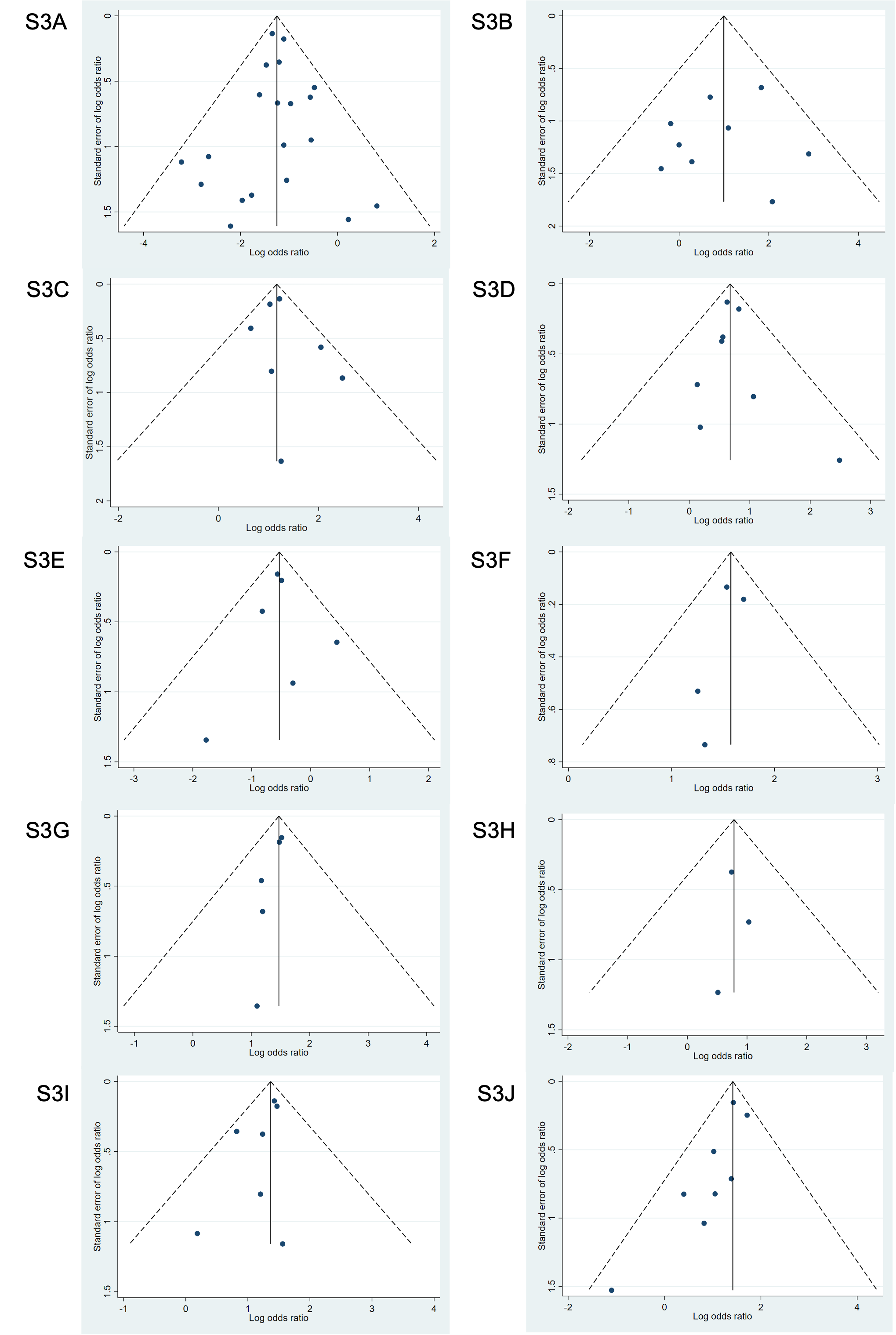

Supplement: Supplement 3 — Funnel plots of publication bias in analyses of (A) primary teratoma, (B) primary seminoma, (C), normal pre-chemotherapy AFP, (D) normal pre-chemotherapy bHCG, (E) normal pre-chemotherapy LDH, (F) greater than or equal to 50% change in mass during chemotherapy, (G) greater than or equal to 70% change in mass during chemotherapy, (H) greater than or equal to 90% change in mass during chemotherapy, (I) residual mass size less than or greater than 2cm, and (J) residual mass size of less than or greater than 5cm for necrosis/fibrosis at pcRPLND. [file Image_3.jpeg]
